# Knee Flexion Strength Before and After ACL Reconstruction Using Hamstring Tendon Autografts

**DOI:** 10.5812/traumamon.12813

**Published:** 2013-10-14

**Authors:** Mohammad Kazem Emami Meybodi, Morteza Jannesari, Alireza Rahim Nia, Habib Yaribeygi, Vahid Sobhani Firoozabad, Ahmad Dorostegan

**Affiliations:** 1 Department of Orthopedics, Baqiyatallah University of Medical Sciences, Tehran, IR Iran; 2 Department of Physiology and Biophysics, Baqiyatallah University of Medical Sciences, Tehran, IR Iran; 3 Sport Physiology Research Center, Baqiyatallah University of Medical Sciences, Tehran, IR Iran

**Keywords:** Knee Flexion Strength, Anterior Cruciate Ligament, Reconstructive Surgical Procedures

## Abstract

**Background:**

Anterior cruciate ligament (ACL) injury is the most common sports injury in both athletes and nonathletes; it can cause disability if not treated correctly. In cases with minor injuries, conservative treatments suffice. But, in cases with ACL tear, surgery by different methods and autografts are indicated. The most prevalent method for ACL reconstruction is the use of hamstring tendon autograft; this requires tendon removal and results in subsequent weakness in patient’s knee flexion strength which can cause dissatisfaction.

**Objectives:**

In this study we evaluate a common procedure used for treating ACL injuries.

**Patients and Methods:**

This study was performed at a hospital in Tehran on 30 patients with ACL tears. Patients’ knee flexion strengths before and 2, 4, 6, and 12 months after reconstruction were measured separately at 20, 45, 90, and 110˚ knee flexion angles, and their means were analyzed using paired t-test.

**Results:**

In this study, knee flexion strength decreased after ACL reconstruction. The greatest decrease in knee flexion strength was observed at 90 and 110˚ knee flexion angles.

**Conclusions:**

Some previous studies have confirmed reduced knee flexion strength following ACL reconstruction at high knee flexion angles. However, some others have denied it. The present study confirmed the reduction in knee flexion strength one year after ACL reconstruction at 90 and 110˚ flexion angles (P = 0.000). Furthermore, the need for physiotherapy, as a process for rehabilitating these patients was also confirmed.

## 1. Background

ACL tearing following knee twisting injuries is common in athletes; it is estimated to be 200,000 cases per year in the United States. About 100000 ACL reconstruction surgeries are performed annually in the United States alone (1). Muscle strengthening has been effective in prevention of ACL tears (2). ACL tear is sometimes asymptomatic and causes no problem for the patient. However, in physically active individuals, it is usually associated with symptoms such as a sense of emptiness along with pain and swelling of the knee. If left untreated, frequent weakness of the knee may lead to meniscus and articular cartilage injuries. Treatment of ACL tears includes reconstruction; which is performed via different techniques. Hamstring tendons (Semitendinosus and Semimembranosus) are routinely used for ACL reconstruction. These tendons are obtained from the patient’s injured lower extremity, prepared and replaced with the torn ACL (3). Another procedure for surgery is the bone-patellar-bone method which is not common today because of its complications after surgery, especially pain in sitting position.

We sought to compare the strength of two-knee flexion in patients after hamstring tendon removal which is involved in knee flexion. Anteromedial bundles of ACL are stronger and contract during knee extension. They prevent the anterior movement of the tibia on femoral condyles during knee extension. The posterolateral bundles of ACL are weaker and are mainly involved in rotational control during knee flexion (4). ACL is comprised of two bundles. Its anteromedial bundles provide anterior stability, while the posterolateral bundles provide rotational stability. The location of femoral tunnel is important in providing this stability, and a vertically oriented femoral canal is among the most common causes of reduced stability following ACL reconstruction (5). ACL reconstruction is performed in subjects younger than 40 years. For older subjects with ACL tear, nonsurgical therapy is recommended that includes modification of sport activities, physiotherapy, and use of a brace. Several studies have reported excellent results for ACL reconstruction in patients older than 40 years (6). Reduction or elimination of the Pivot-shift sign is an important goal in ACL reconstruction. Hamstring muscles which would be removed for ACL reconstruction are among the knee flexors and play a role in knee stability. Strengthening of these muscles somehow plays the same role as ACL. ACL and hamstrings play a similar role in prevention of forward movement of the tibia on femoral condyles (7). If they play a significant role in knee flexion strength, removing them for ACL reconstruction especially in athletes reduces the knee flexion strength and may have unfavorable consequences for the patients. 

## 2. Objectives

The present study aimed to assess the role of two hamstring muscles removed for ACL reconstruction in knee flexion strength and the change in this value after ACL reconstruction using hamstring tendon autograft. If the knee flexion strength is significantly reduced after ACL reconstruction, another method should be used for this purpose. Some studies have failed to show a reduction in knee flexion strength following ACL reconstruction. In a study by Lipscomb in 1995 in the US on 482 patients, no reduction was observed in knee flexion strength after two years of follow up. In their study, the quadriceps muscle strength was also evaluated before and after the procedure. Its post-op strength was 96% of the preoperative value. This is considered as a criterion for good post-op knee physiotherapy. They also compared the knee flexion strength before and after the procedure with that of unoperated knee, and it was observed that during a 2-year follow-up, the flexion strength in the operated knee reached 99% of the unoperated knee (8). 

## 3. Patients and Methods

**Figure 1.**
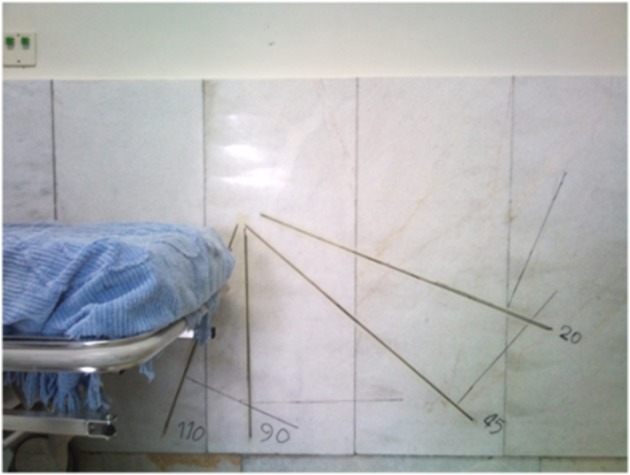
Angles Used to Calculate Knee Strength

**Figure 2.**
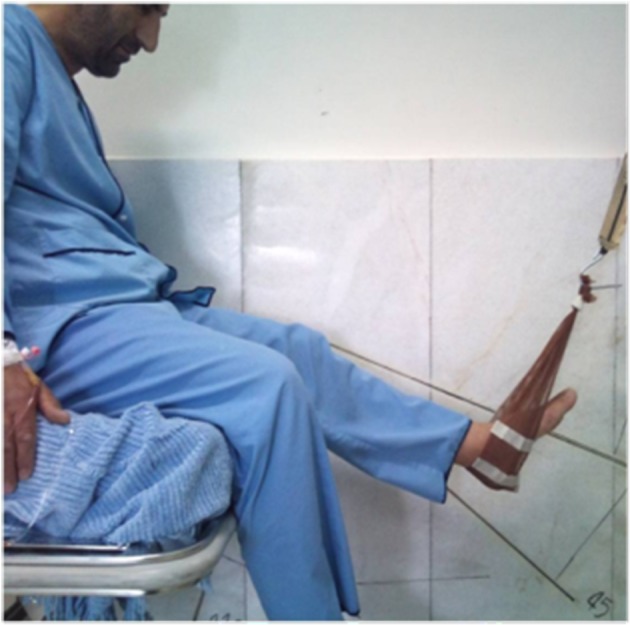
Measurement of Flexion Strength

All patients hospitalized for ACL reconstruction at the Baqiyatallah Hospital from May 20, 2009 to June 19, 2009 (all males with an age range of 21 - 27 years) were evaluated. ACL questionnaire was filled out and a written informed consent was obtained. Before patient selection, patients’ knees were fully examined and they were asked about any history of trauma. None of the patients had any history of trauma except for that causing their ACL tear. None of them had any history of accident or fracture in their lower limbs. Knee examinations such as Lachman test and Anterior Drawer test had positive results for ACL tear. These tests had negative results for the intact knee. Both knees were examined for posteromedial and posterolateral instability. Two patients were excluded from the study due to posterolateral instability. A total of 39 patients were studied. MRI was obtained for the injured knee showing ACL tear in all subjects. Flexion strength of both knees was measured preoperatively at 20, 45 - 90, and 110˚ angles, and at 2, 4, 6, and 12 months in the same position postoperatively. This measurement was performed separately for the injured and intact knees. All patients who underwent ACL reconstruction surgery presented monthly to the orthopedic clinic for routine post-op examinations. Physiotherapy was prescribed for them to strengthen their knee flexor and extensor muscles and improve the knee range of movement to its normal preoperative range (full extension and 130˚ flexion of the knee). Patients presented to the physiotherapy ward for evaluation of the knee flexion strength at the end of the 2nd, 4^th^, and 6th months, and also one year postoperatively. The questionnaire was filled out by a trained technician. The questionnaires were collected after completion of one year follow up. The obtained data was then analyzed and the postoperative knee flexion strength at 2, 4, 6, and 12 months was determined in comparison to the preoperative corresponding values at the same flexion angles (Figure 1 and 2). The knee flexion strength before and after the operation was also evaluated and compared to that of the unoperated knee. Data was analyzed via SPSS software. The frequency of understudy variables was calculated. Paired t-test was used to compare knee flexion strength before and after the operation. 

## 4. Results

Data analysis for 30 patients who regularly showed up for the designated follow up visits (at 2, 4, 6, and 12 months, Table 1) revealed the followings: 

**Frequency:** The frequency of right and left knees ACL rupture were 60% and 40%, respectively. 

**Cause:** ACL injury had occurred in soccer in 53.3%, in martial arts in 26.7%, and in other occasions in 20% of cases.

**Flexion strength (unoperated knee):** The mean unoperated knee flexion strength before the operation was 164 N at 20˚, 180 N at 45˚, 188 N at 90˚, and 176 N at 110˚ flexion angles. The mean unoperated knee flexion strength 6 months after the operation was 161 N at 20˚, 179 N at 45˚, 192 N at 90˚, and 180 N at 110˚ flexion angles. The mean unoperated knee flexion strength 12 months after the operation was 163 N at 20˚, 180 N at 45˚, 189 N at 90˚, and 180 N at 110˚ flexion angles.

**Flexion strength (operated knee):** The mean operated knee flexion strength before the operation was 161 N at 20˚, 179 N at 45˚, 182 N at 90˚, and 174 N at 110˚ flexion angles. The mean operated knee flexion strength 2 months after the operation was 158N at 20˚, 164N at 45˚, 134N at 90˚, and 142N at 110˚ flexion angles. The mean operated knee flexion strength 4 months after the operation was 159 N at 20˚, 172 N at 45˚, 155 N at 90˚, and 142 N at 110˚ flexion angles. The mean operated knee flexion strength 6 months after the operation was 159N at 20˚, 174 N at 45˚, 160 N at 90˚, and 150 N at 110˚ flexion angles. The mean operated knee flexion strength 12 months after the operation was 164N at 20˚, 172N at 45˚, 167N at 90˚, and 161N at 110˚ flexion angles.

**Analyses: **The mean operated knee flexion strength 2 months after the surgery decreased by 2% compared to its preoperative value at 20˚ (P = 0.199), by 8.5% at 45˚, by 26% at 90˚ (P = 0.000), and by 30% at 110˚ flexion angles (P = 0.000). The mean operated knee flexion strength 4 months after the surgery compared to its preoperative value decreased by 1.5% at 20˚ angle (P = 0.119), 4% at 45˚ angle (P = 0.000), 14.5% at 90˚ angle (P = 0.000), and 18% at 110˚ flexion angle (P = 0.000). The mean operated knee flexion strength 6 months after the surgery compared to its preoperative value decreased by 1.5% at 20˚ angle (P = 0.123), 3% at 45˚ angle (P = 0.005), 22% at 90˚ angle (P = 0.000), and 13% at 110˚ flexion angle (P = 0.000). The mean operated knee flexion strength 12 months after the surgery compared to its preoperative value decreased by 1.5% at 20˚ angle (P = 0.187), 4% at 45˚ angle (P = 0.013), 8% at 90˚ angle (P = 0.000), and 7% at 110˚ flexion angle (P = 0.000). The mean operated knee flexion strength compared to that of unoperated knee 12 months after the surgery increased by 0.5% at 20˚ angle, and decreased by 4% at 45˚ angle, 12% at 90˚ angle, and 10% at 110˚ flexion angle. The mean unoperated knee flexion strength 12 months postoperatively compared to its preoperative value decreased by 1% at 20˚ angle (P = 0.200), was equal to the preoperative value at 45˚ angle (P = 0.956), increased by 0.3% at 90˚ angle (P = 0.733), and increased by 2% at 110˚ flexion angle (P = 0.037).

One year postoperation, the mean knee flexion strength compared to its preoperative value did not significantly decrease at 20˚ and 45˚ angles, but showed a significant reduction at higher knee flexion angles (90˚ and 110˚).

**Table 1.** The Mean Knee Flexion and Extension Strength at Different Time Points and Angles

Strength at Different Follow up Times, N	Strength at Different Angles			
	20˚	45˚	90˚	110˚
**Mean unoperated knee flexion strength preoperatively**	164.93	180	188.73	176.10
**Mean unoperated knee flexion strength 6 months postoperatively**	161.17	179.33	192	180.10
**Mean unoperated knee flexion strength 12 months postoperatively**	163.40	180.07	189.33	180.00
**Mean operated knee flexion strength preoperatively**	161.70	179.33	182.10	174.00
**Mean operated knee flexion strength 2 months postoperatively**	158.77	164.20	134.20	122.07
**Mean operated knee flexion strength 4 months postoperatively**	159.40	172.10	155.37	142.77
**Mean operated knee flexion strength 6 months postoperatively**	159.43	174.00	160.00	150.67
**Mean operated knee flexion strength 12 months postoperatively**	164.00	172.67	167.33	161.33

None of the patients developed infection, knee pain, or knee emptiness 6 months postoperatively. All patients underwent physiotherapy (in the first 2 months for 20 sessions) and muscle strengthening at home. None of them went back to their previous sport activity. The mean age of patients was 25 years. There were 12 soldiers and 18 executive officers. By increasing the knee flexion angle, the mean post-op knee flexion strength suffered a greater reduction compared to its preoperative value. This reduction was statistically significant. One year postoperatively, the mean knee flexion strength compared to its preoperative value did not show a significant reduction at 20˚ and 45˚ angles, but a significant reduction was noted at higher knee flexion angles (90˚ and 110˚).

## 5. Discussion

We concluded that the knee flexion strength decreases after the operation and the magnitude of this reduction increases at higher knee flexion angles. Physiotherapy of the operated knee gradually improves the knee flexion strength and approximates the preoperative value. Eventually, the knee flexion strength value may equal the preoperative value at 20 and 45˚ angles one year after the operation. However, at 90˚ and 110˚ angles, the mean knee flexion strength was still lower than the corresponding preoperative value at the one year follow up. This difference was statistically significant (P = 0.000 at 90˚, P = 0.000 at 110˚). Some studies have confirmed our findings but some others have stated otherwise. Our study results are in contrast to those of Lipscomb et al. (9), Kazunouri et al. (10), and Carter et al. (11). The mentioned studies did not report any reduction in knee flexion strength after ACL reconstruction and rehabilitation. However, our study findings were in accordance with the results of Viola et al. (12), and Tadokoro et al. (9). Our study confirmed the theory of reduction of knee flexion strength at high flexion angles (90˚ and 110˚) after ACL reconstruction surgery using hamstring tendons. This reduction is important in some sports like weightlifting. In such cases, ACL reconstruction is recommended to be performed through another technique. Future studies are required to assess the effect of reduction in knee flexion strength on the performance of athletes. 

A significant reduction in flexion strength of the operated knee one year postoperation and after the completion of rehabilitation period compared to the control group indicates that removing hamstring muscles for ACL reconstruction may be harmful in athletes especially the professionals who need the strength of these muscles for their physical activity. Another technique should be used for ACL reconstruction in such cases.
